# Displacements prediction from 3D finite element model of maxillary protraction with and without rapid maxillary expansion in a patient with unilateral cleft palate and alveolus

**DOI:** 10.1186/s12938-015-0074-9

**Published:** 2015-08-19

**Authors:** Dan Zhang, Li Zheng, Qiang Wang, Li Lu, Jia Ma

**Affiliations:** Department of Orthodontics, School of Stomatology, China Medical University, Shenyang, China; School of Material Science and Engineering, Shenyang University of Technology, Shenyang, China; Department of Dental Materials, School of Stomatology, Key Lab of Liaoning Province, China Medical University, Shenyang, China; Department of Oral and Maxillofacial Surgery, School of Stomatology, China Medical University, 117# Nanjing North Street, Heping District, Shenyang, 110002 Liaoning China

**Keywords:** Three-dimensional finite element analysis, Maxillary protraction, Rapid maxillary expansion, Unilateral cleft palate and alveolus

## Abstract

**Background:**

Both maxillary protraction and rapid expansion are recommended for patients with cleft palate and alveolus. The aim of the study is to establish a three-dimensional finite element model of the craniomaxillary complex with unilateral cleft palate and alveolus to simulate maxillary protraction with and without rapid maxillary expansion. The study also investigates the deformation of the craniomaxillary complex after applied orthopaedic forces in different directions.

**Methods:**

A three dimensional finite element model of 1,277,568 hexahedral elements (C3D8) and 1,801,945 nodes was established based upon CT scan of a patient with unilateral cleft palate and alveolus on the right side in this study. A force of 4.9 N per side was directed on the anatomic height of contour on the buccal side of the first molar. The angles between the force vector and occlusal plane were −30°, −20°, −10°, 0°, 10°, 20°, and 30°. A force of 2.45 N on each loading point was directed on the anatomic height of contour on the lingual side of the first premolar and the first molar to simulate the expansion of the palate.

**Results:**

The craniomaxillary complex displaced forward under any of the loading conditions. The sagittal and vertical displacement of the craniomaxillary complex reached their peak at the protraction degree of −10° forward and downward to the occlusal plane. There were larger sagittal displacements when the maxilla was protracted forward with maxillary expansion. The palatal plane rotated counterclockwise under any of the loading conditions. Being protracted without expansion, the dental arch was constricted. When supplemented with maxillary expansion, the width of the dental arch increased. Transverse deformation of the dental arch on affected side was different from that on unaffected side.

**Conclusions:**

Protraction force alone led the craniomaxillary complex moved forward and counterclockwise, accompanied with lateral constrain on the dental arch. Additional rapid maxillary expansion resulted in a more positive reaction including both larger sagittal displacement and the width of the dental arch increase.

## Background

Cleft palate and alveolus are common congenital anomalies that are receiving much attention from stomatologists [1–4]. Children who have a cleft palate usually need surgery at around 6 months of age to repair the cleft and they often display different degrees of midfacial hypoplasia after surgery [5–7]. As patients grow, occlusion, speech, breathing, swallowing and aesthetics, among other things, may be affected if the patient did not receive appropriate treatment for long periods of time [8–10]. Orthodontists often provide maxillary protraction for children in the growing and developing periods that suffer from cleft palate and alveolus to stimulate maxillary [11, 12]. Some investigations have manifested skeletal, alveolar and dental effects from short-term maxillary protraction. Forward displacements of the maxilla, clockwise rotation of the mandible, protrusion of the upper incisors and retrusion of the lower incisors are seen [13, 14], but the skeletal effects of maxillary protraction remain controversial. There are no significant skeletal changes in some long-term follow-ups [13, 15].

In addition to the maxillary deficiency of patients with cleft palate and alveolus in the sagittal direction, bone defect and the injury of the surgery causes deficiency in the transverse direction [16–21]. Maxillary protraction therapy is commonly supplemented with rapid maxillary expansion for these patients [22–25]. Some evidence in the literature suggests that maxillary expansion alone can be beneficial in the treatment of certain types of Class III malocclusion [26, 27]. These investigations have also shown that the direction of the force is critical in controlling the rotation of the maxilla. Nanda and Hickory noted [28], based on the functional matrix theory, that the maxillary growth pattern was similar to the effect of maxillary protraction, and the displacement pattern of the maxillary complex can be altered by the direction of the traction force.

Finite element analysis is a mathematical method in which the shape of complex geometric objects and their physical properties are computer constructed. Interactions of various components of the model are then calculated for stress, strain, and deformation. Among which, the displacement changes of the objects is the most intuitive evaluation indicators. Three-dimensional (3D) finite element method was first used in orthodontics by Thresher and Saito [29] to study stresses on human teeth. Ever since, this method has proved effective in many dental fields such as simulation of tooth movement and optimization of orthodontic mechanics.

The aim of the study is to establish a 3D finite element model of the craniomaxillary complex with unilateral cleft palate and alveolus to simulate maxillary protraction with and without rapid maxillary expansion. The study also investigates the displacement changes of the craniomaxillary complex after applied orthopedic forces in different directions.

## Methods

### Patient data and craniomaxillary complex reconstruction

Ethical approval of this research was obtained from the ethics committee of China Medical University. The scan data was obtained from a 13-year-old boy with unilateral cleft palate and alveolus on the right side. Written informed consent was obtained from his parents. Spiral computed tomography images (SomatonLightspeed/64 plus; GE Healthcare, Waukesha, WI, USA) of the craniofacial complex region (0.5-mm layer; voxel size, 0.44 × 0.44 × 0.5 mm^3^) were obtained, saved as digital imaging and communications in medicine (DICOM) files, and then imported into Mimics^®^ software (Materialise, Leuven, Belgium) for model reconstruction. A Hounsfield unit (HU) with a value range of 226–3071 was used to identify and distinguish craniomaxillary bone from other tissues. The craniomaxillary complex was segmented from the skull by using the “erase” function in the software on each slice of the data set.

**Fig. 1:**
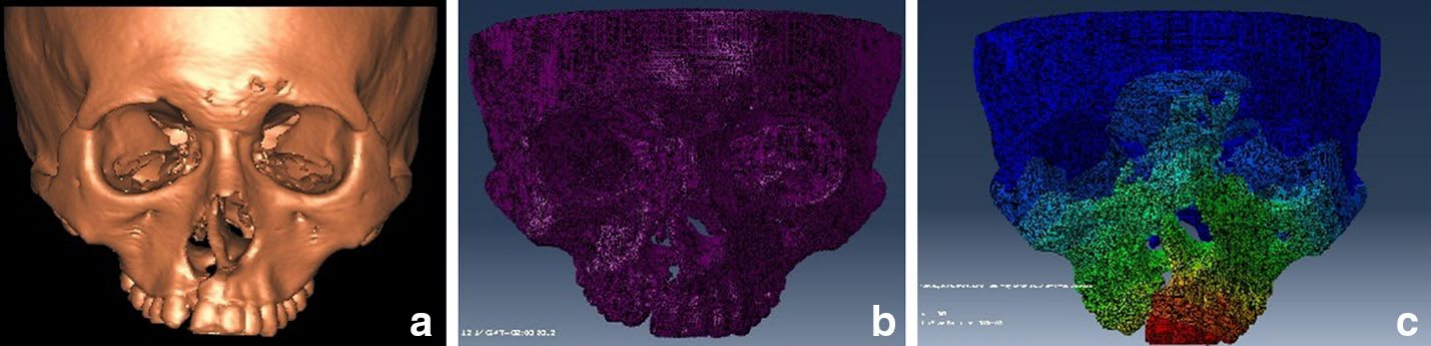
The craniomaxillary complex after **a** 3D construction, **b** mesh generation and **c** FEM analysis

The reconstructed geometry of craniomaxillary complex was exported in stereolithography (STL) format (Fig. 1).

### Computational grid of craniomaxillary complex

The STL file was imported into MSC. Marc^®^ (MSC Software Corporation, Santa Ana, CA, USA), which was used to generate a volume mesh from the 3D geometry of the craniomaxillary complex. The craniomaxillary complex was meshed into 1,277,568 hexahedral elements (C3D8) and 1,801,945 nodes (Fig. 1).

### Material properties, boundary and loading conditions

The meshed model was then exported into Abaqus software (ABAQUS Inc., Providence, RI, USA). The outermost elements of the craniofacial bone were supposed as the compact bones. Finally, a 3D finite element model consisted of 628,228 elements for the cortical bones, 620,087 elements for the cancellous bones, and 29,253 elements for the teeth was established. The mechanical properties of the cortical and cancellous bones and teeth in the model (Table 1) were defined based on the experimental data from previous studies [30–34]. Materials in the analysis were assumed to be linearly elastic and isotropic.

**Table 1:** Young’s modulus and Poisson’s ratio for the materials used in this study (Iseri et al. [30], Jafari et al. [31], Pan et al. [32], Lee et al. [33] and Xiulin Yan et al. [34])

Material	Young’s modulus (N/mm^2^)	Poisson’s ratio
Compact bone	1.37 × 10^4^	0.3
Cancellous bone	7.9 × 10^3^	0.3
Tooth	2.0 × 10^4^	0.3

**Fig. 2:**
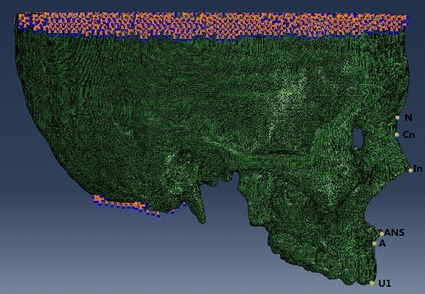
Boundary conditions of the craniomaxillary complex and marker nodes on the anterior of the craniomaxillary complex

Zero-displacement and zero-rotation boundary conditions were imposed on the nodes along the foramen magnum, and restraints were established at all other nodes of the cranium lying on the symmetrical plane (Fig. 2).

**Fig. 3:**
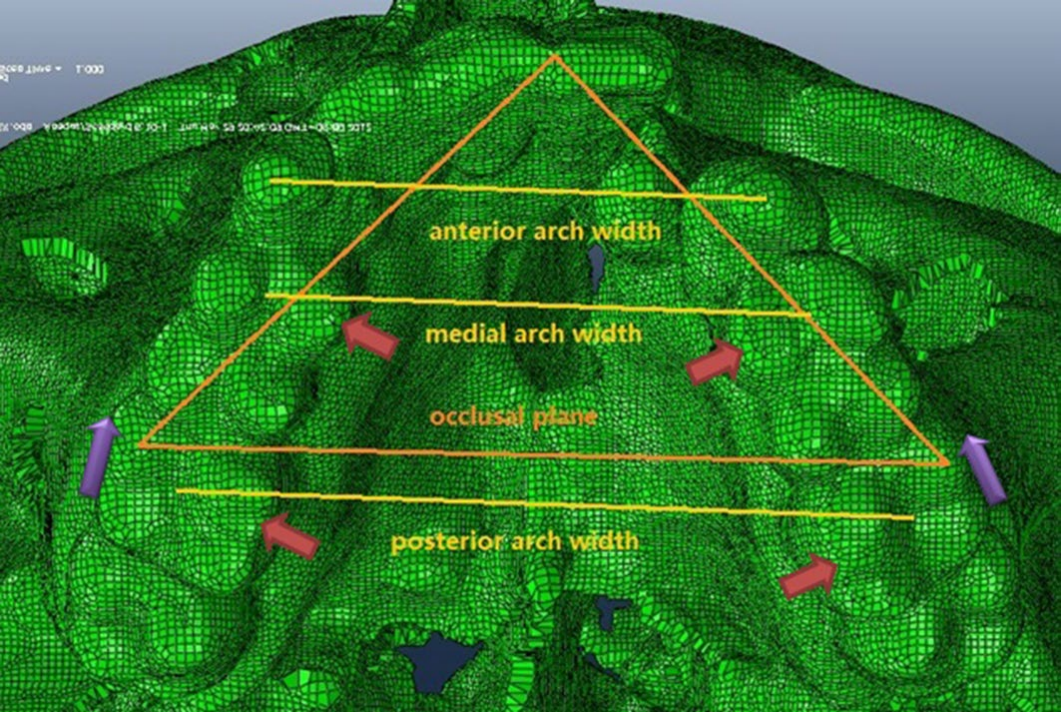
Loading conditions and the width of the maxillary dental arch

In this study, a force of 4.9 N per side was directed forward on the anatomic height of contour on the buccal side of the first molar. The angles between the force vector and occlusal plane were −30°, −20°, −10°, 0°, 10°, 20°, and 30°. A force of 2.45 N on each loading point was directed on the anatomic height of contour on the lingual side of the first premolar and the first molar to simulate the expansion of the palate (Fig. 3). So there were totally 14 loading cases in this study.

To measure the amount of displacement of the craniomaxillary complex, representative nodes in the frontal, sagittal and transverse planes were selected. First, the amount of sagittal displacement of the craniomaxillary complex, which was generally used when comparing the effect of an orthopaedic appliance following maxillary protraction [34, 35] was measured at eight marker nodes in the sagittal plane. These included four dental marker nodes and four skeletal marker nodes. Among these nodes, subspinale (A), upper incisor (U1), left first molar’s palatal cusp tip (ML1) and right first molar’s palatal cusp tip (MR1) represent the characteristics of the dentition and the alveolar bone, and nasion (N), the most concave part of the nasal bone (Cn), inferior part of the nasal bone (In) and anterior nasal spine (ANS) represent the skeletal characteristics of the craniomaxillary complex. The detailed marker location was shown in Fig. 2. Second, the vertical displacement of the complex was analyzed by measuring the vertical displacement of ANS and posterior nasal spine (PNS) in the palatal plane. Lastly, the width of the dental arch was measured. Anterior arch width was analyzed by measuring the distance between the cusps of the canine. Width of the medial dental arch was analyzed by measuring the distance between the central fossa of the first premolar. The distance between the central fossa of the fist molar was measured to analyze the width of the posterior dental arch (Fig. 3).

## Results

**Fig. 4:**
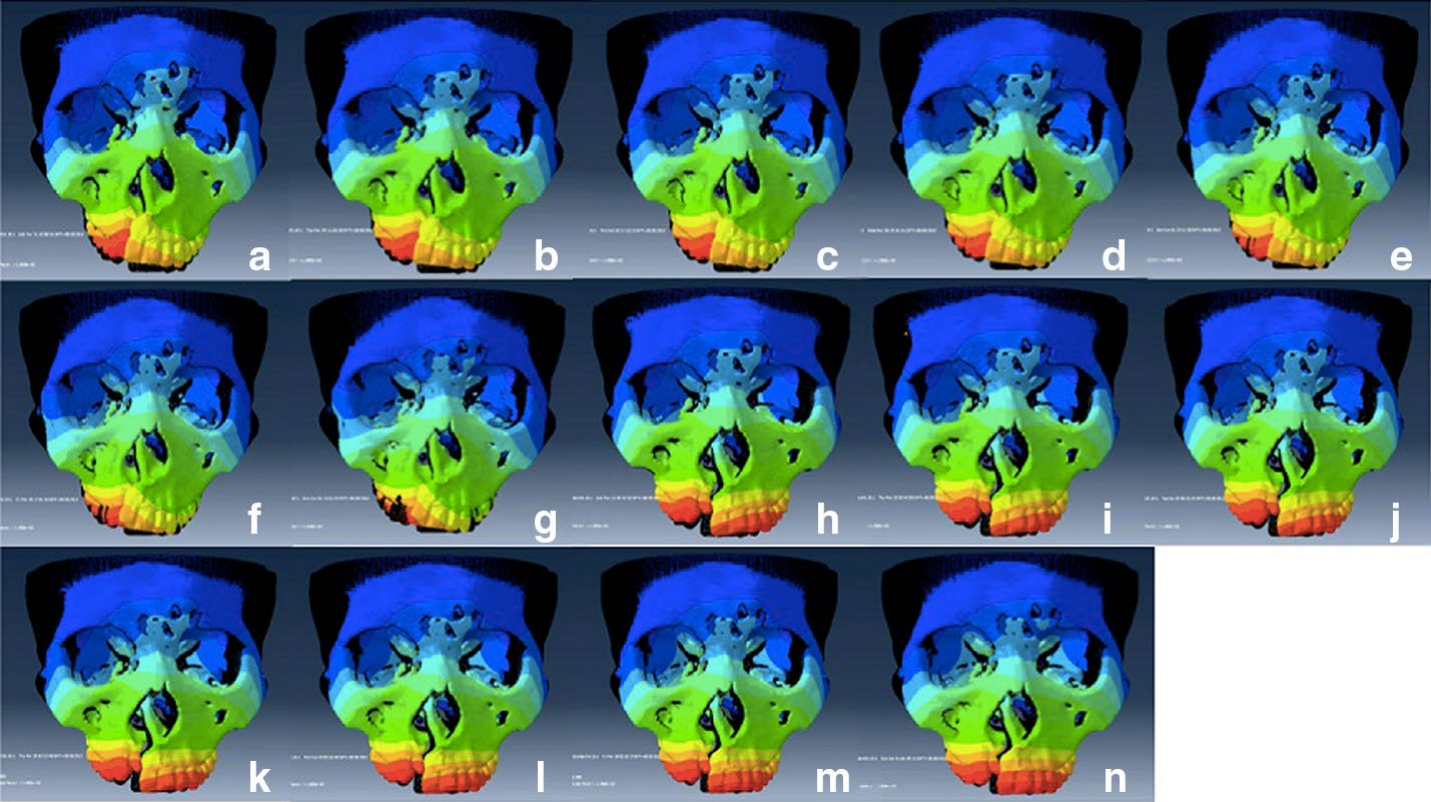
Superimposed contours of displacement under different loading conditions with the same coordinate system and magnification factor. **a** −30° protraction without maxillary expansion; **b** −20° protraction without maxillary expansion; **c** −10° protraction without maxillary expansion; **d** 0° protraction without maxillary expansion; **e** 10° protraction without maxillary expansion; **f** 20° protraction without maxillary expansion; **g** 30° protraction without maxillary expansion; **h** −30° protraction supplemented with maxillary expansion; **i** −20° protraction supplemented with maxillary expansion; **j** −10° protraction supplemented with maxillary expansion; **k** 0° protraction supplemented with maxillary expansion; **l** 10° protraction supplemented with maxillary expansion; **m** 20° protraction supplemented with maxillary expansion; **n** 30° protraction supplemented with maxillary expansion. *Scale factor* 100

The deformed maxillae under different loading conditions and the original ones were overlapped in Fig. 4. Both with and without rapid maxillary expansion, the front of the craniomaxillary complex moved upward in any of the loading conditions. The vertical displacement of the maxilla was decreased with the help of expansion at the same protraction degree. When the maxilla was protracted forward without expansion, the alveolus cleft was constricted. In the situation of maxillary expansion, the alveolus cleft was expanded. In both of the situation, the width of the cleft decreased gradually with the increase of the protraction degree.

### The sagittal displacements of 4 dental marker nodes at different protraction degrees

**Fig. 5:**
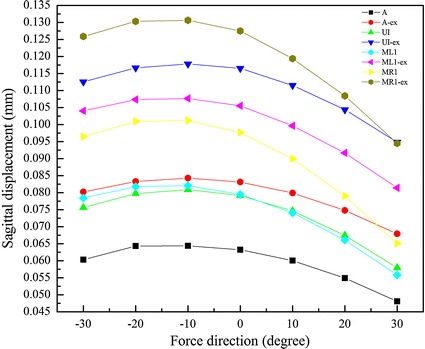
The sagittal displacements of four dental marker nodes (A, UI, ML1 and MR1)

**Fig. 6:**
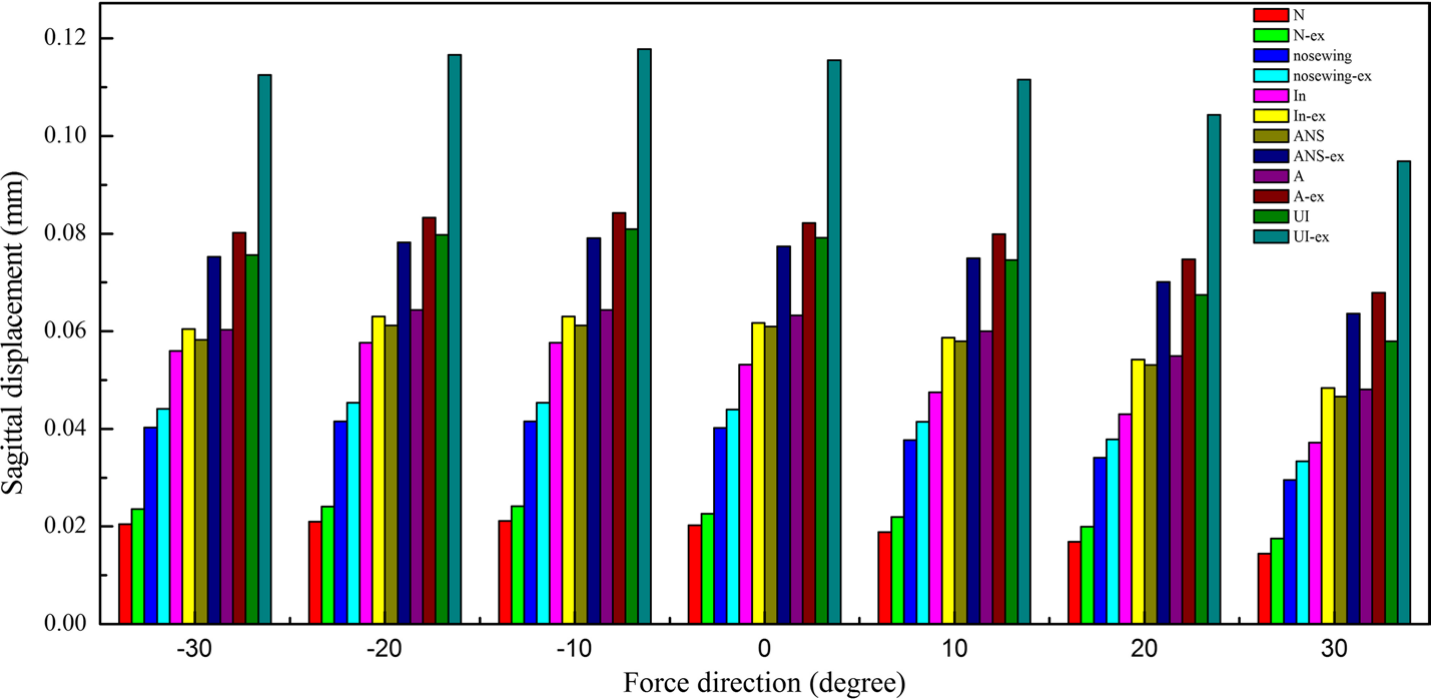
The sagittal displacements of six marker nodes on the anterior craniomaxillary complex (N, Cn, In, ANS, A and UI)

The sagittal displacements of A, UI, ML1 and MR1 at different protraction degrees are shown in Figs. 5 and 6. It is clear that both with and without rapid maxillary expansion, the marker nodes displaced forward in any of the loading conditions. The maximum displacement was obtained at the protraction degree of −10°. In the situation of maxilla expansion, the sagittal displacements of the marker nodes were larger than those of maker nodes without maxillary expansion at the same protraction degree. In both of the situation, the displacement of UI is larger than that of A in any of the loading conditions, as is that of MR1 (affected side) compared to ML1 (unaffected side).

### The sagittal displacement of 4 skeletal marker nodes at different protraction degrees

The sagittal displacements of N, Cn, In and ANS at different protraction degrees are show in Fig. 6. With and without rapid maxillary expansion, the skeletal nodes displaced forward in any of the loading conditions. The sagittal displacement of the selected nodes increased gradually from superior to inferior of the craniomaxillary complex and peaked at −10° protraction.

Figure 6 also shows the sagittal displacements of the dental marker nodes and the skeletal marker nodes on the anterior of the craniomaxillary complex. At the protraction degree of −10° forward and downward to the occlusal plane, the sagittal displacement of the maxilla reached the maximum. At the same time, the sagittal displacements of the selected nodes increased gradually from superior to inferior of the craniomaxillary complex.

### The vertical displacement of the palatal plane at different protraction degrees

**Fig. 7:**
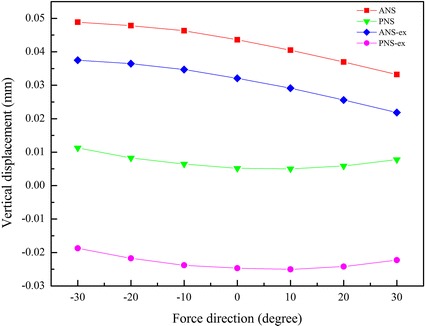
The vertical displacement of the palatal plane

**Fig. 8:**
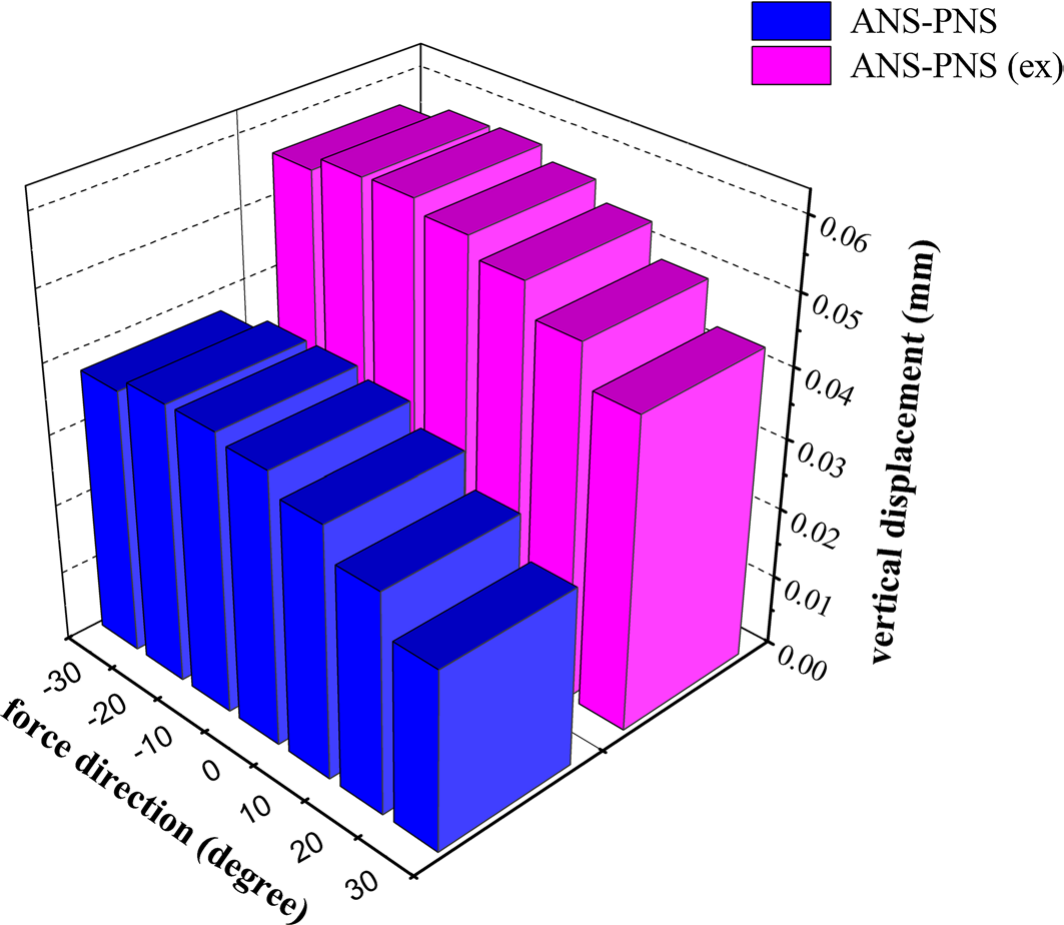
The rotational amplitude of the palatal plane

The vertical displacements of the palatal plane at different protraction degrees are show in Figs. 7 and 8. Both with and without rapid maxillary expansion, the vertical displacements of ANS had positive values, as the anterior of the palatal plane moved upward under any of the protraction degrees. In the situation of non-expansion, the vertical displacement of ANS was larger than that in the situation of maxillary expansion at the same protraction degree. In the situation of non-expansion, the vertical displacements of PNS had positive values, indicating that the posterior palatal plane moved upward. In contrast, the posterior palatal plane moved downward when the maxillary was protracted with expansion. The palatal plane rotated counterclockwise under any of the loading conditions, and the rotational amplitude peaked at −10° protraction.

### The changes of the dental arch width

The variation of the maxillary dental arch width was shown in Table 2. When maxilla was protracted forward without expansion, the dental arch narrowed down with the increase of the protraction degree. The transverse deformation of the arch width increased from posterior to anterior. With the supplement of rapid maxillary expansion, the dental arch was expanded. The transverse deformation of the arch width decreased gradually with the increase of the protraction degree.

**Table 2:** Deformation of the dental arch width under different protraction degrees

Protraction degree	Anterior arch (mm)	Medial arch (mm)	Posterior arch (mm)
−30°	−0.0676	−0.0547	−0.0333
−20°	−0.0702	−0.0568	−0.0342
−10°	−0.0745	−0.0609	−0.0374
0°	−0.0799	−0.0663	−0.0424
10°	−0.0860	−0.0728	−0.0488
20°	−0.0928	−0.0801	−0.0564
30°	−0.1001	−0.0882	−0.0652
−30°(ex)	0.0532	0.0534	0.0480
−20°(ex)	0.0507	0.0513	0.0468
−10°(ex)	0.0463	0.0472	0.0447
0°(ex)	0.0398	0.0409	0.0416
10°(ex)	0.0348	0.0353	0.0365
20°(ex)	0.0280	0.0280	0.0289
30°(ex)	0.0208	0.0199	0.0201

Negative values if the dental arch width decreased, positive values if the dental arch width increased

**Fig. 9:**
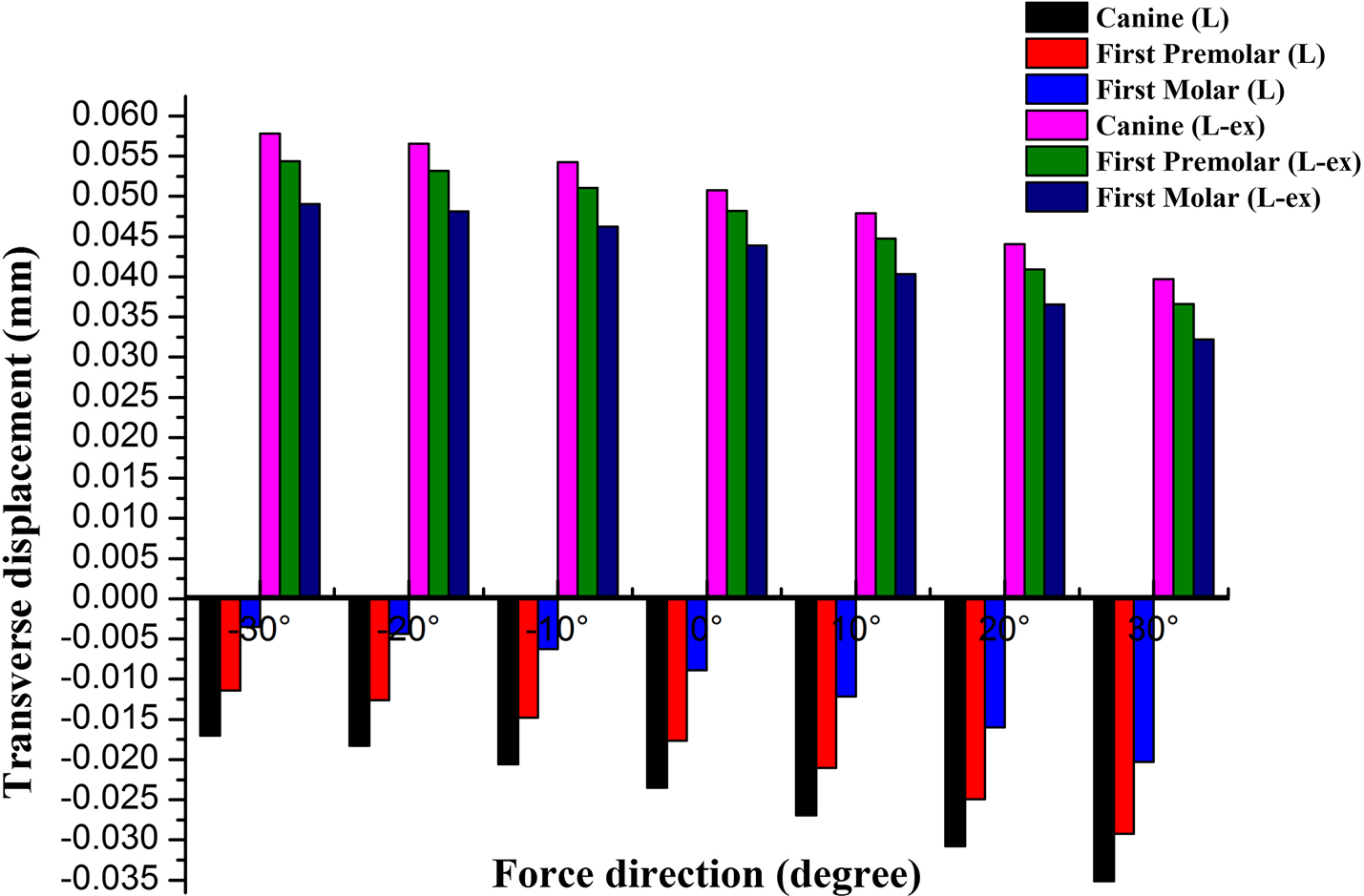
The transverse displacement of the dental arch on the unaffected side. *Negative values* if the dental arch moved to the *right*. *Positive values* if the dental arch moved to the *left*

**Fig. 10:**
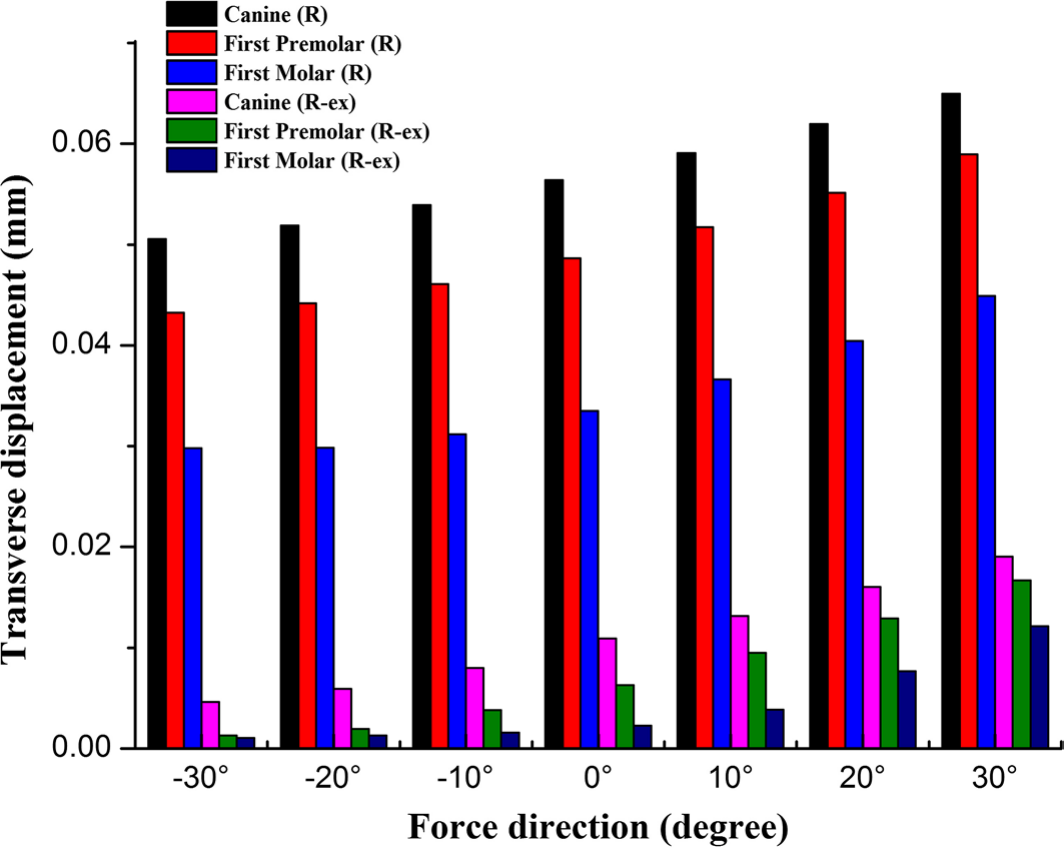
The transverse displacement of the dental arch on the affected side. *Negative values* if the dental arch moved to the *right*. *Positive values* if the dental arch moved to the *left*

Transverse deformation of the dental arch was shown in Figs. 9 and 10. The dental arch on the unaffected side (the left side) was constricted and moved to the right (negative values) by the protraction of the maxilla. With the supplement of maxilla expansion, the dental arch was expanded and moved to the left (positive values). The dental arch on the affected side (the right side) was constricted and moved to the left (positive values) in both of the situations. But, the expansion of the dental arch would greatly reduce the tendency of constriction. In both situations, the deformation of the dental arch increased gradually from posterior to anterior at the same protraction degree. With the increase of protraction degree, the tendency of constriction increased, while the tendency of expansion decreased gradually.

## Discussion

In cleft palate and alveolus patients, the mandible is usually unaffected by the cleft and grows normally, however, the maxilla often does not grow as far forward and downward as the non-cleft child, resulting in a short maxilla and a Class III malocclusion [36]. The scar contracture that occurs from the hard palate repair is thought to distort the growth of the maxilla resulting in maxillary hypoplasia [16–21]. Maxillary protraction is recommended for cleft palate and alveolus patients with skeletal maxillary deficiency [5, 28, 37, 38]. The principle of maxillary protraction is to apply tensile force on the craniomaxillary sutures and thereby stimulate bone apposition in the suture areas. Numerous animal experiments have shown that a maxillary protraction appliance with controlled force is effective on anterior displacement and bone formation at the cartilaginous suture area of the maxillary complex [39, 40].

Maxillary deficiency in patients with cleft palate and alveolus occurred in both the sagittal and the transverse direction due to the bone defect and the injury of the surgery. Therefore, maxillary protraction therapy is often supplemented with maxillary expansion to correct the insufficient maxillary arch width [16, 41]. Rapid maxillary expansion is typically used in young patient and has been shown to help correct the sagittal defect of the maxilla [42, 43]. Haas [44, 45] reported the orthopedic effect of rapid maxillary expansion, which produced a forward and downward tipping of the maxilla with concomitant downward and backward mandibular rotation. These orthopedic changes facilitated the correction of a mild deficiency of the midface. Turley [46] stated that palatal expansion “disarticulates” the maxilla and initiates cellular responses in these circumaxillary sutures allowing a more positive reaction to protraction forces. Melsen [47] confirmed these increased cellular responses to rapid maxillary expansion. The displacement of various craniofacial structures was considerably more after maxillary protraction with maxillary expansion. This agrees with Yu et al. [35], who showed greater displacement in the frontal, vertical and lateral directions with maxillary expansion when compared with no maxillary expansion. In this study, both with and without rapid maxillary expansion, the maxilla moved forward under any of the loading conditions. The sagittal displacement of the maxilla was greater with the help of maxillary expansion. At the protraction degree of −10° forward and downward to the occlusal plane, the sagittal displacement of the maxilla reached the maximum. At the same time, the sagittal displacements of the selected nodes increased gradually from superior to inferior of the craniomaxillary complex. Therefore, the protraction of the maxilla causes more dental than skeletal changes. For patient with flared upper incisors, the difference in the dental and skeletal displacement will affect the protrusion of the upper lip.

It is necessary to control vertical growth during maxillary protraction in patients with cleft palate and alveolus, which has been reported as difficult. Lower anterior facial height may decrease, remain the same, or even increase during maxillary protraction [48–56]. Possible changes in lower anterior facial height during maxillary protraction under forces from various directions may play an important role in the prognosis of patients with a Class III malocclusion and maxillary deficiency [57–59]. Biomechanical studies were performed to explore the relationship between the direction of applied force and the displacement of the craniomaxillary complex by using the finite element method, which is a helpful and reliable mathematical instrument in orthodontics [58–60].

Merwin et al. [60] and Ngan et al. [61] reported that the counterclockwise rotational tendency of the maxilla was the reason for contraindication of maxillary protraction therapy in Class III patients with maxillary deficiency and open bite. In this study, regardless of whether maxillary protraction was supplemented with or without rapid maxillary expansion, the palatal plane rotated counterclockwise in all loading conditions. The palatal plane rotational amplitude of the expansion group was larger than that of the non-expansion group at the same protraction degree. Therefore, maxillary protraction, especially supplemented with rapid maxillary expansion, should be used with caution for cleft palate and alveolus patients with maxillary deficiency and open bite.

The rotational amplitude peaked at −10° protraction and decreased gradually on both sides. The sagittal displacement of the anterior of craniomaxillary complex also peaked at −10° protraction. The above results show that the maximum value of the forward displacement and the counterclockwise of the palatal plane can be obtained at the protraction degree of −10°. Therefore, a protraction direction of −10° is recommended for patients with maxillary deficiency and deep bite. In addition, the supplement with rapid maxillary expansion can greatly improve the above effect.

Bone graft of the alveolus cleft would be obtained in patients during periods of growth and development when the canines are erupting [62]. Therefore, the width alternation of the dental arch by the protraction of the maxilla is one of the main concerns of orthodontics and oral surgeons.

Lei et al. [63] found that the dental arch of cleft lip and palate patient tended to be constricted after maxillary protraction. As it has been shown in Table 2, the dental arch was constricted after maxillary protraction. With the supplement of maxillary expansion, the width of the dental arch increased. As it has been shown in this study, the transverse deformation of the affected side was different from that of the unaffected side. The dental arch was constricted by the protraction of the maxilla. With the supplement of maxillary expansion, the dental arch on the unaffected side was expanded while the dental arch on the unaffected side was still constricted. But, the expansion of the dental arch would greatly reduce the tendency of constriction on the affected side. With the increase of protraction degree, the tendency of constriction increased. So, an asymmetric expansion would be suitable for patients with unilateral cleft palate and alveolus. The use of a counterclockwise protraction force increases its efficiency. Lei et al. [63] found that the tendency for constriction at the anterior region of the dental arch was one of the most important side effects for maxillary protraction. It has also been found in this study, the deformation of the dental arch increased gradually from posterior to anterior at the same protraction degree. In all the situations, alternation of the dental arch width was more sensitive in the anterior part of the dental arch than the posterior part of the dental arch.

The anatomy of the midface is complex, as the maxilla articulates with ten other facial bones and with the anterior and middle cranial base. Therefore, a refined finite element model is needed for precise and realistic simulation. Although tetrahedral elements have commonly been used in biomechanical applications [58, 64, 65] because automated meshing techniques are available, hexahedral elements offer attractive numerical properties relative to tetrahedrons [66, 67]. Benzley et al. compared the accuracy of the hexahedral meshes with the tetrahedral meshes in their study, found that linear hexahedrons could generally deform in a lower strain energy state, thus making them more accurate than linear tetrahedrons in numerous situations [68]. Xiulin Yan et al. reported that tetrahedral elements are not as accuracy and reliable as hexahedral elements for finite element models with complicated geometry [34]. In this study, an element model consisting of 1,277,568 hexahedral elements (C3D8) and 1,801,945 nodes individually was established, which greatly increase the accuracy of the model. The results of this study may differ from data obtained from in vivo investigations. Additional biochemical and clinical studies, as well as animal experiments, are needed to understand the effects of maxillary protraction with and without the use of rapid maxillary expansion in more detail. Progressive research with clinical identification of dynamic modeling is also required to investigate the effects of protraction, both with and without the use of maxillary expansion on the facial musculature and other soft tissues.

## Conclusions

1. The craniomaxillary complex moved forward and counterclockwise under a protraction force −30° to 30° forward and downward to the occlusal plane.
2. Rapid maxillary expansion provided a more positive reaction to maxillary protraction. Greater deformation was obtained by maxillary expansion when compared with no maxillary expansion.
3. At the protraction degree of −10° forward and downward to the occlusal plane, sagittal deformation of the maxilla reached the maximum. The palatal plane rotated counterclockwise under any of the loading conditions, and the rotational amplitude peaked at −10° protraction.
4. At the same protraction degree, sagittal deformation of the maxilla increased gradually from superior to inferior.
5. When maxilla was protracted forward without expansion, the dental arch narrowed down with the increase of the protraction degree. The transverse deformation of the arch width increased from posterior to anterior. With the supplement of rapid maxillary expansion, the dental arch was expanded. The transverse deformation of the arch width decreased gradually with the increase of the protraction degree.
6. The dental arch on the unaffected side was constricted by the protraction of the maxilla and would be expanded by maxillary expansion. But, the dental arch on the affected side was constricted in both of the situations. With the increased of the protraction degree, the constricted displacement increased gradually.

The clinical interpretation of this study may be as follows:

1. Maxillary protraction, especially supplemented with rapid maxillary expansion is recommended for patients with maxillary deficiency.
2. A protraction direction of −10° is recommended for patients with maxillary deficiency and deep bite. In addition, the supplement with rapid maxillary expansion can greatly improve the above effect.
3. Asymmetric expansion is recommended for patients with unilateral cleft palate and alveolus. The use of a counterclockwise protraction force may enhance its efficiency.
